# Detection of *Peste des petits ruminants* virus and goatpox virus from an outbreak in goats with high mortality in Meghalaya state, India

**DOI:** 10.14202/vetworld.2016.1025-1027

**Published:** 2016-09-28

**Authors:** A. Karim, U. Bhattacharjee, K. Puro, I. Shakuntala, R. Sanjukta, S. Das, S. Ghatak, A. Sen

**Affiliations:** Division of Animal Health, Indian Council of Agricultural Research - North Eastern Hill Region, Umiam - 793 013, Meghalaya, India

**Keywords:** Capripoxvirus, disease, goat, mortality, *Peste des petits ruminants*

## Abstract

**Aim:**

We describe a laboratory investigation carried out to confirm the etiology of the heavy mortality (37 animals died out of total 44, i.e. 84%) in goats in Ri-Bhoi district of Meghalaya, Northeast region of India in December 2015. The clinical signs observed were abortion, diarrhea, high fever (up to 104°F), pox lesion in the skin, and respiratory distress.

**Materials and Methods:**

The samples comprising whole blood, sera, and pox lesion were collected from the animals (n=7) from an outbreak for the screening of *peste des petits ruminants* (PPR) and poxviruses. The whole blood and sera were used for screening of PPR virus (PPRV) by sandwich enzyme-linked immunosorbent assay (ELISA) and antibody by competitive ELISA as well as detection of PPRV partial N gene by reverse transcription-polymerase chain reaction (PCR). The skin lesions were used for the detection of poxvirus by PCR.

**Results:**

The results showed the presence of PPR antigens (58-80%) in the samples by sandwich ELISA and antibody in all the sera samples ranging from 9% to 41% positivity in competitive ELISA. Four samples were positive for PPRV partial N gene. The skin lesion screened for poxvirus was also found to be positive for I3L gene of goatpox virus.

**Conclusion:**

We confirm the outbreak of disease in goats with high mortality is a case of mixed infection of PPR and goatpox detected for the first time in Northeast India.

## Introduction

*Peste des petits ruminants* (PPR) or goat plague is an acute or subacute viral disease of sheep and goat characterized by severe pyrexia, oculonasal discharges, necrotizing and erosive stomatitis, enteritis, and pneumonia [1]. It is caused by a morbillivirus of family Paramyxoviridae which is related to rinderpest, measles, and canine distemper. The disease causes high morbidity and mortality in susceptible sheep and goats. Goats are more susceptible than sheep [2].

PPR is enzootic in India and outbreaks occur regularly among small ruminants throughout the country [3,4]. The outbreak of PPR was reported in January 2014 in Nongsder village, Ri-Bhoi district of Meghalaya with 22% mortality (40 animals died out of 180 affected) [5]. The world organization for animal health has identified PPR as a notifiable and economically important transboundary viral disease of sheep and goats associated with high morbidity and mortality [6,7]. Goatpox (Capripox) is also endemic in India [8]. Goatpox is caused by strains of *Capripoxvirus* and produces a characteristic clinical disease in fully susceptible breeds of sheep and goats. It causes high mortality and morbidity, and they incur severe economic threat to poor farmers [9]. In India, outbreaks of sheeppox and goatpox have been reported regularly [10]. Dual infection of PPR and goatpox was recorded in indigenous goat of central India [11].

Here, we report the occurrence of mixed infection of PPR and goatpox detected for the first time in Northeast India causing high mortality.

## Materials and Methods

### Ethical approval

As per CPCSEA guidelines, studies involving clinical samples does not require approval of Institute Animal Ethics Committee. However, samples were collected as per standard sample collection methods without any harm or stress to the animals.

### Outbreak investigation

An outbreak was investigated in a goat farm in Paham Mawlein village, Ri-Bhoi District in Meghalaya, in December 2015. A total of 44 numbers of goats in the age group of 5 months were brought from four villages of neighboring Assam, *viz*., Hojai, Dhubri, Nagaon, and Kamrup in September 2015. The animals started developing clinical signs of unthriftiness, less feed intake. The affected animals were showing diarrhea, high fever, pox lesion in the skin, and respiratory distress. Abortion was also noticed in pregnant animals, and 37 animals died out of total 44 animals, i.e., 84% mortality was recorded. There was no record of vaccination, but the animals were dewormed. The outbreak was suspected of PPR with goatpox. The whole blood, sera, and pox lesion samples were collected from the animals remaining (n=7) for the screening of PPR and poxviruses.

The whole blood and sera were used for screening of PPR virus (PPRV) and screened by competitive enzyme-linked immunosorbent assay (ELISA) using commercial kits for the detection of anti-PPRV nucleoprotein antibodies in serum (ID screen^®^ PPR competition) and sandwich ELISA for PPRV (ID screen^®^ PPR Antigen Capture) following manufacturer’s instructions. The absorbance was read at 492 nm with an ELISA reader (Lab systems Multiskan Plus, Thermo Fisher Scientific, USA). The result was calculated as sample positivity (S/N) for antibodies in percentage (%). If S/N ≤ 50% - positive, 50-60%- doubtful and ≥ 60% - negative and the sample positivity (S/N) for antigen in percentage (%) was if S/N ≤ 20% - negative and ≥ 20% - positive.

Molecular detection of PPR-specific genes by reverse transcription-polymerase chain reaction (RT-PCR) was done [12] using whole blood to detect partial N genes with the primer sequence forward - ACAGGCGCAGGTCTCCTTCCT and reverse - TGATTTCCACAGAGGGTG. The skin pox lesion was triturated, and 10% suspension was made with phosphate-buffered saline (pH 7.4). Detection of poxvirus was done by targeting I3L gene as reported Venkatesan *et al*. [10] by PCR with the primer sequences forward - GATATAGAATAGGGCTAGTTGCAG and reverse - CATCAAAAATGACATCTACATATATAGC for goatpox virus and forward - GCCAGGAACTTTATATTCGATG with the same reverse for sheeppox virus. RT-PCR was done with total RNA extracted from blood samples using a QIAamp viral RNA mini kit (Qiagen, GmbH, Hilden, Germany). Extracted RNA was reverse transcribed using a RevertAid first-strand cDNA synthesis kit (Thermo Scientific, USA) employing a random hexamer primer following manufacturers’ protocol. The synthesized cDNA was used for PPRV detection. The DNA from the tissue was extracted using QIAamp viral DNA kit (Qiagen, GmbH, Hilden, Germany) and used for detection of poxviruses. The PCR reaction was performed in a thermal cycler (Eppendorf, Hamburg, Germany) in 50 μL volume containing 1 μg DNA sample, 10 pmol of each forward and reverse primer, 5 μL of the ×10 buffer (100 mM Tris-HCl pH 8.8, 500 mM KCl, 0.8% NP-40), 2 mM JMgCl_2_, 200 μM/μL of each dNTPs, and 1 U of JumpStart™ Taq DNA polymerase (M/s Sigma, Foster City, CA, USA). Amplification was performed by initial denaturation at 95°C for 5 min followed by 35 cycles of denaturation at 94°C for 30 s, annealing at 55°C for 35 s for partial N gene and 63°C 30 s for I3L gene and extension at 72°C for 30 s with a final extension at 72°C for 7 min. Amplicons were analyzed by electrophoresis through 1.5% agarose - Tris-acetate - ethylenediaminetetraacetic acid (EDTA) (40 mM Tris-acetate, pH 8.0, 1 mM EDTA) gel, stained with ethidium bromide and visualized in gel documentation system (DNR MiniLumi, Israel).

## Results and Discussion

The ELISA results showed the presence of anti-PPRV nucleoprotein antibodies in all the sera samples (n=7) ranging from 9% to 41% positivity in competitive ELISA. The PPRV was also detected by sandwich ELISA with positivity ranges from 58% to 80% indicating varying viral load of individual animals. From the whole blood, four samples were positive for PPRV N gene by RT-PCR. In a study conducted on seroprevalence of PPR in North East India during 2013-14, Balamurugan *et al*. [13] reported the overall seroprevalence of 17.90% in suspected animals and 11.63% in random sampling. The outbreak of PPR in Nongsder village in January 2014 was recorded 22% mortality [5]. In the present case, the heavy mortality observed (84%) is possibly due to the occurrence of mixed infection of PPR and goatpox. The skin lesion screened by PCR for poxvirus was also found to be positive for I3L gene of goatpox virus. Goatpox and contagious ecthyma were recorded as sporadic in the northeast, but the dual infection of PPR and goatpox was first time reported in the state. The occurrence of dual infection of PPR and goatpox in indigenous goats was earlier reported in central India [11]. It warrants appropriate control measures since goatpox virus can be of threat [14] and exhibits change in host specificity and pathogenesis [9,15]. Furthermore, PPR is enzootic in India and regular outbreaks were reported among small ruminants throughout the country incurring significant economic losses in terms of morbidity, mortality, and loss of productivity due to trade restriction [2]. Since 1994, a number of PPR outbreaks have been reported in different states of India with variable morbidity and mortality rates [16], and PPR outbreaks have been linked to the introduction of new animals into flocks since the animals are usually under stress due to traveling over long distances [13]. During their migration, these animals frequently infect local populations along the migration route and may be one of the reasons for the higher frequency of PPR outbreaks with increased susceptibility [2]. The infected animals help to maintain viral circulation throughout the year via frequent animal to animal transmission [14]. In the present case, the animals were transported from the neighboring state with no record of prevalence or incidence of disease in the area. The infection might be from the point of origin or exposed en route. The location of the present outbreak is within 100 km radius of the previous outbreak of PPR. However, chances of animal to animal transmissions are remote due to the difference in animal husbandry practices in the region and geographical locations. Therefore, effective surveillance and monitoring is required for establishing control strategies of important animal diseases.

## Conclusion

We confirm the outbreak of disease in a goat with high mortality is due to mixed infection of PPR and goatpox. The prevalence of mixed infection is detected and reporting for the first time in Meghalaya, India. It warrants appropriate control measures since these viruses can be of threat to the livestock of the region. Effective surveillance and monitoring will be the key in establishing control strategies of these important animal diseases.

## Authors’ Contributions

An outbreak was reported to AS and IS for investigation. They planned and tasked the work to others. AK, SD, and RS went for field investigation and collection of samples. AK and SG did the serological work. UB and KP did the molecular work. AK and KP drafted and revised the manuscript. All authors read and approved the final manuscript.
